# A transcriptional analysis of carotenoid, chlorophyll and plastidial isoprenoid biosynthesis genes during development and osmotic stress responses in *Arabidopsis thaliana*

**DOI:** 10.1186/1752-0509-5-77

**Published:** 2011-05-19

**Authors:** Stuart Meier, Oren Tzfadia, Ratnakar Vallabhaneni, Chris Gehring, Eleanore T Wurtzel

**Affiliations:** 1 Division of Chemistry, Life Science and Engineering, King Abdullah University of Science and Technology, Thuwal 23955-6900, Kingdom of Saudi Arabia; 2 Department of Biological Sciences, Lehman College, The City University of New York, 250 Bedford Park Blvd. West, Bronx, NY 10468, USA; 3 The Graduate School and University Center-CUNY, 365 Fifth Ave., New York, NY 10016-4309, USA; 4 Department of Biotechnology, University of the Western Cape, Private Bag X17, Cape Town - Bellville 7535, South Africa

## Abstract

**Background:**

The carotenoids are pure isoprenoids that are essential components of the photosynthetic apparatus and are coordinately synthesized with chlorophylls in chloroplasts. However, little is known about the mechanisms that regulate carotenoid biosynthesis or the mechanisms that coordinate this synthesis with that of chlorophylls and other plastidial synthesized isoprenoid-derived compounds, including quinones, gibberellic acid and abscisic acid. Here, a comprehensive transcriptional analysis of individual carotenoid and isoprenoid-related biosynthesis pathway genes was performed in order to elucidate the role of transcriptional regulation in the coordinated synthesis of these compounds and to identify regulatory components that may mediate this process in *Arabidopsis thaliana*.

**Results:**

A global microarray expression correlation analysis revealed that the phytoene synthase gene, which encodes the first dedicated and rate-limiting enzyme of carotenogenesis, is highly co-expressed with many photosynthesis-related genes including many isoprenoid-related biosynthesis pathway genes. Chemical and mutant analysis revealed that induction of the co-expressed genes following germination was dependent on gibberellic acid and brassinosteroids (BR) but was inhibited by abscisic acid (ABA). Mutant analyses further revealed that expression of many of the genes is suppressed in dark grown plants by Phytochrome Interacting transcription Factors (PIFs) and activated by photoactivated phytochromes, which in turn degrade PIFs and mediate a coordinated induction of the genes. The promoters of *PSY *and the co-expressed genes were found to contain an enrichment in putative BR-auxin response elements and G-boxes, which bind PIFs, further supporting a role for BRs and PIFs in regulating expression of the genes. In osmotically stressed root tissue, transcription of Calvin cycle, methylerythritol 4-phosphate pathway and carotenoid biosynthesis genes is induced and uncoupled from that of chlorophyll biosynthesis genes in a manner that is consistent with the increased synthesis of carotenoid precursors for ABA biosynthesis. In all tissues examined, induction of β-carotene hydroxylase transcript levels are linked to an increased demand for ABA.

**Conclusions:**

This analysis provides compelling evidence to suggest that coordinated transcriptional regulation of isoprenoid-related biosynthesis pathway genes plays a major role in coordinating the synthesis of functionally related chloroplast localized isoprenoid-derived compounds.

## Background

The carotenoids are pure isoprenoids that are synthesized in chloroplasts from geranylgeranyl diphosphate (GGPP) which additionally serves as an immediate precursor for other chloroplastic localized isoprenoid biosynthesis pathways including plastoquinone (PQ), the phytol tail of chlorophylls, phylloquinones (PhQ) and tocopherols as well as the phytohormone gibberellic acid (GA). While the biochemistry of carotenoid biosynthesis (CrtBS) has been extensively studied and most genes encoding enzymes that function in the CrtBS pathway have been identified, little is known about how the synthesis of these enzymes is coordinated and additionally how this synthesis is coordinated with that of other interdependent and interrelated isoprenoid-derived compounds. We have performed a global *in-silico *expression correlation analysis using microarray experimental data to identify genes that share a high level of co-expression and thus may share closely associated functional relationships with phytoene synthase (*PSY*). Comprehensive expression profiling of chloroplastic isoprenoid-related biosynthesis pathway genes was performed over a range of developmental and stress-related conditions in order to identify important regulatory components such as phytohormones and transcriptional regulatory factors that are important in coordinating their collective expression.

**Figure 1:**
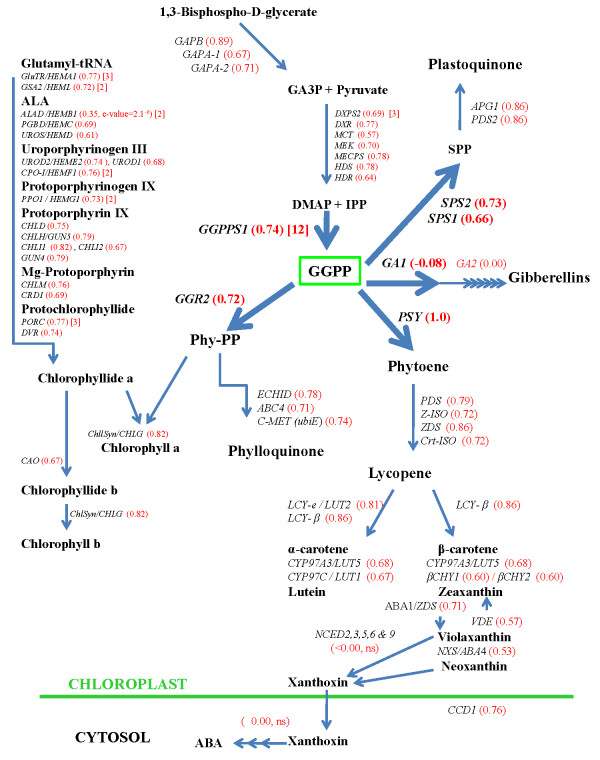
**Diagram of the plastidial isoprenoid biosynthesis pathway detailing the level of co-expression that *PSY *shares with individual interrelated isoprenoid biosynthesis pathway genes**. The pathways represented include the Calvin Cycle, MEP, Carotenoid, Chlorophyll, Phylloqinone, Plastoquinone, ABA and Gibberellins and are collectively referred to as the *PSY*-correlated isoprenoid interrelated genes (*PSY-CIIG*). Reaction substrates and products are represented in bold black letters while genes that encode pathway enzymes are in black italic letters. Numbers in red parentheses represent expression correlation r-values and numbers in square brackets indicate the number of paralog genes that are annotated to encode the respective enzymes. All r-values > 0.5 had p-values and e-values <1.0^-15^. Non-significant r-values are indicated as n.s. Only the highest correlated member of the paralog gene family and those that have a co-expression value > 0.6 are listed. See Additional File 1 for list of corresponding gene IDs, details of statistics for individual genes and an extended list including additional paralog gene family members.

A number of chloroplast localized isoprenoid-derived compounds constitute important components of the photosynthetic apparatus. The carotenoids perform a range of functions including the acquisition of light energy and photoprotection [1] and additionally serve as precursors for abcisic acid (ABA) biosynthesis [2]. The chlorophylls are the main light absorbing pigments of the photosynthetic apparatus while PhQ and PQ function in photosynthetic electron transfer reactions. Plastoquinone additionally functions as an essential electron carrier in CrtBS desaturation reactions mediated by phytoene desaturase (PDS) and ζ-carotene desaturase (ZDS) [3]. As the GGPP molecule is an immediate precursor for the biosynthesis of these functionally related molecules, it serves as an important metabolic hub in the biosynthesis of essential components of the photosynthetic apparatus (see Figure 1, dark text).

The synthesis of GGPP in plastids starts from pyruvate and glyceraldehyde 3-phosphate (GAP), that can be generated directly from the Calvin cycle (photosynthesis) or glycolysis [4], and serve as precursor molecules for the methylerythritol 4-phosphate (MEP) pathway [5]. The MEP pathway consists of a series of seven enzymes that function sequentially to catalyze the synthesis of the prenyl diphosphate precursors, isopentenyl diphosphate (IPP) and dimethylallyl diphosphate (DMAPP). GGPP synthase (GGPPS) then catalyzes the sequential addition of three molecules of IPP to one molecule of DMAPP resulting in the formation of the poly-isoprenoid, GGPP [4].

The *PSY *gene encodes the first dedicated enzyme of the CrtBS pathway which catalyzes the condensation of two molecules GGPP to form 15-*cis*-phytoene (Figure 1) [6-8]. Phytoene is metabolized to lycopene in a linear series of desaturation and isomerisation reactions that involves four enzymes [9,10]. CrtBS then branches into two distinct pathways (Figure 1), the β-cyclase (LCY-β, also known as LCYB or LYC-β) enzyme converts lycopene into β-carotene while the dual action of epsilon cyclase (LCY-ε, also known as LCYE or LYC-ε) and LCY-β results in the formation of α-carotene. The α- and β- carotenes can then be hydroxylated to form α- and β- branch xanthophylls which are essential components of the photosynthetic apparatus in higher plants where they function in photosystem assembly, light harvesting and photoprotection [11,12]. In addition, violaxanthin and neoxanthin also serve as direct precursors for ABA biosynthesis and are alternative substrates for plastid localized nine *cis*-epoxycarotenoid dioxygenases (NCEDs) [2,13] (Figure 1).

The carotenoids have been shown to have important functional roles in early stages of post-germination development. In etioplasts of dark grown seedlings, lutein and violaxanthin biosynthesis is required for assembly of the prolamellar body (PLB) [14,15]; a lattice of tubular membranes composed primarily of lipids, carotenoids and a ternary complex of NADPH, protochlorophyllide oxidoreductase (POR) and the chlorophyll precursor, protochlorophyllide (Pchlide) [16]. The synthesis of carotenoids in PLBs is thought to optimize the transition of etiolated plants to photomorphogenic development since it has been shown to enhance chlorophyll accumulation and greening upon light-induced de-etiolation [14,17].

Light activates the differentiation of etioplasts into chloroplasts in a process that is accompanied by a large and coordinated increase in the biosynthesis and accumulation of carotenoids, chlorophylls and pigment-binding proteins; this accumulation supports the development of a functional photosynthetic apparatus [14,15,18]. The light-induced synthesis of carotenoids is characterized by an increase in expression of *PSY *and select MEP pathway genes [18,19] as well as an increase in PSY enzymatic activity [15]. The expression of *PSY *has been shown to be elevated in response to a broad spectrum of continuous (c) light wavelengths including far-red (cFR), red (cR), blue (cB) and white (cW) [18,20,21]. While *PSY *transcript levels have been reported to increase in response to cFR [18], only light wavelengths that activate POR - which catalyzes the light-dependent conversion of Pchlide to chlorophyllide - cause the decay of PLBs, the synthesis of chlorophylls and the transition of etioplasts into chloroplasts [15,22]. These studies demonstrate that the coordinated and co-localized synthesis of carotenoids with chlorophyll precursors and chlorophylls in etiolated and de-etiolated plants respectively is required for normal photomorphogenic development.

The light-induced increase in *PSY *expression has been shown to be mediated by the phytochrome (PHYs) photoreceptors. Mutant studies have shown that the induction of *PSY *expression in response to cFR is dependent on the light-labile PHY-A while the cR-induction is thought to be mediated by light-stable PHYs other than PHY-B [18]. Upon light-induced activation, the cytoplasmic localized PHYs are translocated to the nucleus where they interact with and mediate the degradation of the Phytochrome Interacting transcription Factors (PIFs); these factors bind to G-boxes in the promoters of light-induced genes and negatively regulate their expression [23]. Recently, the PIFs have been shown to have an important role in regulating the transcription of *PSY *and other carotenoid and chlorophyll biosynthesis genes during light-induced de-etiolation [24]. The PHYs and PIFs are therefore interesting candidate regulatory factors that may function to coordinate the transcription of genes that encode enzymes that function in the interrelated and interdependent chloroplastic isoprenoid biosynthesis pathways during early development.

Transcriptional co-regulation has been shown to play a major role in coordinating cellular responses that involve multiple genes and their products. A number of studies have shown that genes that have been confirmed to be co-expressed in response to a range of conditions have correlated functional relationships, including physical interactions between their encoded proteins [25-28]. These finding also extend to metabolic pathways where it has been shown that many genes encoding metabolic enzymes that function within the same or functionally related pathways form co-expression modules [29,30]. Thus, it is conceivable, that the synthesis of functionally related chloroplast localized isoprenoid molecules is mediated by their transcriptional co-regulation.

The model plant species *Arabidopsis thaliana *is ideal for studying global transcriptional responses since there are thousands of publicly available full-genome microarray experiments that encompass a broad range of experimental conditions including different developmental stages, stress, chemical and hormone treatments and mutants. In addition, analysis tools are available to identify modules of co-expressing genes and genome sequence data allows analysis of promoter regulatory regions and the identification of putative regulatory elements.

It is pertinent to acknowledge that changes in gene transcription do not necessarily translate to changes in protein abundance and functional activity due to post-transcriptional regulatory mechanisms. However, as these mechanisms rely on a gene being transcribed in the first instance, gene transcription can be considered the primary level of regulation of protein synthesis. While cells can alter the activity of specific proteins/enzymes to fine tune cellular responses, the protein must be synthesized and present at appropriate quantities for this to occur. Changes in gene transcription in response to specific stimuli can be considered a primary regulatory response that reflects a change in requirement for a specific protein(s) at a specific point in time. In addition, in comparison to a single gene, when the expression of a large group of functionally related genes is altered in a uniform manner in response to a specific stimulus, it is a stronger indicator that the transcriptional response is representative of a cell's intent to change the associated functional activity in response to the stimuli.

Here we aim to elucidate the role transcriptional regulation plays in coordinating CrtBS and the synthesis of other functionally related isoprenoid-derived compounds during early development and in response to osmotic stress. A global co-expression analysis revealed that *PSY *is highly co-expressed with many photosynthesis-related genes including, those involved in chlorophyll, PQ and PhQ biosynthesis as well as genes that function in the upstream Calvin cycle and MEP pathway that synthesize the commonly required GGPP precursor. Stimulus-specific transcription profiling revealed that expression of the isoprenoid biosynthesis genes is almost universally activated following germination, during both etiolated and de-etiolated growth and the induction during early development is positively regulated by BRs and GA and inhibited by ABA. During etiolated growth, the PIFs appear to suppress the expression of the genes while PHYs mediate their photoactivation. An enrichment in putative BR-auxin response elements and G-boxes (which bind PIFs) in the promoter of *PSY *and the co-expressed genes further supports a role for BRs and PIFs in regulating expression of the genes. In osmotically stressed root tissue, transcription of CrtBS-related genes is induced in a manner that is consistent with the increased synthesis of carotenoid precursors for ABA biosynthesis. In all tissues examined, induction of β-carotene hydroxylase transcript levels is linked to increased demand for ABA. We therefore conclude that transcriptional regulation plays a major role in coordinating the synthesis of functionally related isoprenoid-derived compounds in chloroplasts.

## Results and Discussion

### PSY co-expression analysis

In order to elucidate the role transcriptional regulation plays in coordinating CrtBS and the synthesis of other functionally related isoprenoid-derived compounds; an expression correlation analysis was undertaken using *PSY *as the driver gene in order to determine the level of co-expression that *PSY *shares with all of the other genes represented on the ATH1 microarray (22 K) chip. Key to the accuracy of this analysis is that co-expression is measured over a large number of diverse experimental conditions (see methods and Ref [31]). *PSY *was selected as the driver gene for this analysis as it is the first dedicated enzyme of carotenogenesis and its transcription is known to be positively correlated with and a major driving force for carotenoid production [9,14,20,32]. It is thought that genes that are highly co-expressed with *PSY *will have closely associated functional roles.

The expression of *PSY *was shown to be highly correlated with many genes in the genome with the top 50 expression correlated genes (*PSY-ECG50*) having a Pearson correlation coefficient (r-value) ranging from 0.91 to 0.84 (Table 1). In total, approximately 1000 genes (4.3%) had an r-value > 0.6 while around 600 (2.6%) had an r-value > 0.7 supporting the specificity of the analysis since it indicates that *PSY *is co-expressed with only small percentage of select genes in the *Arabidopsis thaliana *genome. All genes in the *PSY-ECG50 *had highly significant p-values (<1^-35^) and e-values (<1^-35^) supporting the biological significance of the results.

**Table 1:** List of the 50 genes that are most highly co-expressed with *PSY*

ID	r	DESCRIPTION	GO
AT5G17230	1	Phytoene synthase (PSY)	^CMP, Pd, CPl^

AT2G04039	0.910	Expressed protein (ExPr)	^Pd, CPl^

AT1G62750	0.902	Snowy cotyledon 1 (SCO1), Elongation factor Tu	^CO, Pd, CPl, TF^

AT1G14345	0.898	Transmembrane domain, oxidoreductase	^Pd, PP, TP, CPl^

AT1G16880	0.897	Uridylyltransferase-related	^AB, TS, Cd, Pd, PP, TP, CPl^

AT3G55330	0.896	Photosystem II reaction center PsbP family protein (PPL1)	^PS, Pd, PP, TP, CPl^

AT1G55480	0.896	Similar to LPA1 (Low PSII accum1),	^Pd, PP, TP, CPl^

AT1G09340	0.890	Chloroplast RNA binding (CRB)	^AB, CO, TS, Cd, Cd, Pd, PP, CPl^

AT1G26220	0.890	GCN5-related N-acetyltransferase (GNAT) family protein	

AT1G42970	0.889	Glyceraldehyde-3-phosphate dehydrogenase B subunit (GAPB)	^PS, AB, TS, Cd, PM, PSD, Pd, PP, TP, CPl^

AT4G34090	0.888	ExPr//chloroplast stroma	

AT1G50320	0.887	Thioredoxin X (ATHX)	^Pd, CPl^

AT1G54500	0.887	Rubredoxin family protein	^PM, Pd, PP, TP, CPl^

AT1G17220	0.882	Fu-gaeri1 (FUG1), Translation initiation factor IF-2, chloroplast	^TF^

AT5G44650	0.881	ExPr//chloroplast thylakoid membrane	^Pd, PP, TP, CPl^

AT3G26570	0.881	Phosphate transporter 2;1 (PHT2;1)	^Pd, PP, CPl^

AT5G04140	0.881	Glutamate synthase 1 (GLU1)/ferredoxin-dependent	^AB, Pd, CPl, OR^

AT4G01800	0.877	Preprotein translocase secA subunit, chloroplast [precursor]	

AT1G11860	0.876	Aminomethyltransferase, mitochondrial precursor	

AT1G45474	0.874	Photosystem I light harvesting complex gene 5 (LHCA5)	^PS, PM, PSL, TP^

AT1G73110	0.874	Ribulose bisphosphate carboxylase/oxygenase activase, putative	^Pd, PP, TP, CPl^

AT2G21330	0.873	Fructose-bisphosphate aldolase 1 (FBA1)	^PM, Pd, PP, CPl, CF^

AT5G58260	0.873	Encodes subunit NDH-N of NAD(P)H:plastoquinone dehydrogenase	^Pd, PP, TP, CPl^

AT5G43750	0.871	NAD(P)H dehydrogenase 18 (NDH18)	^Pd, PP, TP, CPl^

AT1G15980	0.870	NDH-dependent cyclic electron flow 1 (NDF1)	^Pd, CPl^

AT4G10300	0.869	ExPr	^Pd, CPl^

AT5G17170	0.867	Rubredoxin family protein, enhancer of sos3-1 (ENH1)	^PM, Pd, PP, TP, CPl^

AT3G04790	0.866	Ribose 5-phosphate isomerase-related	^PS, PM, PSD, Pd, PP, TP, CPl, CF^

AT1G05140	0.866	Membrane-associated zinc metalloprotease	^Pd^

AT5G08650	0.866	GTP-binding protein LepA, putative	^Pd, CPl, TF^

AT5G23120	0.865	High chlorophyll fluorescence 136 (HCF136) PS II assembly,	^Pd, PP, TP, CPl^

AT1G32470	0.865	Glycine cleavage system H protein, mitochondrial precursor	^OR^

AT1G01320	0.865	Tetratricopeptide repeat (TPR)-containing protein l	

AT1G32080	0.864	Membrane protein, putative contains 12 transmembrane domains	^Pd, PP, CPl^

AT2G20890	0.863	Thylakoid formation1 (THF1)	^PS, PM, PSL, Pd, PP, TP, CPl^

AT3G11950	0.862	Phytoene desaturation 2 (PDS2), UbiA prenyltransferase	

AT1G18060	0.862	ExPr	

AT3G54050	0.862	Fructose-1,6-bisphosphatase, putative	^AB, TS, Cd, PM, Pd, PP, CF^

AT3G10230	0.862	Lycopene cyclase (LCY-β)	^CMP, Pd, CPl,^

AT2G34860	0.861	Embryo sac development arrest 3 (EDA3), Heat shock protein 40	^Pd, CPl^

AT1G27480	0.860	Lecithin:cholesterol acyltransferase family protein (LACT)	

AT3G63410	0.860	Albino or pale green mutant (APGM), MPBQ methyltransferase	^Pd, PP, CPl^

AT1G07010	0.860	Calcineurin-like phosphoesterase family protein	^Pd, CPl^

AT1G76450	0.858	Oxygen-evolving complex-related	^Pd, PP, TP, CPl^

AT5G42310	0.858	PPR repeat-containing protein	

AT3G04870	0.857	Zetacarotene desaturase (ZDS)	^CMP^

AT1G77090	0.856	Thylakoid lumenal 29.8 kDa protein i	^PS, Pd, PP, TP, CPl^

AT1G64680	0.856	ExPr	

AT1G80030	0.855	DNAJ heat shock protein,	^Pd, PP, TP, CPl^

AT4G17600	0.855	Light-harvesting-like protein (Lil3:1)	^Pd, PP, TP, CPl^

AT5G08050	0.854	ExPr	

An expression correlation analysis was performed to identify genes in the Arabidopsis genome that are most highly co-expressed with PSY. Co-expression is measured as expression correlation (r-value). Genes in the top 50 expression correlated genes (*PSY-ECG50*) that were found to belong to a functionally enriched category are indicated. NOTE: The r-values listed for all genes are highly significant (p-values < 1^-35 ^and e-values <1^-35^). ExPr, expressed protein. **GO biological process**: PS = photosynthesis (L3), AB = response to abiotic stimulus (L3), TS = response to temperature stimulus (L4), PM = generation of precursor metabolites and energy (L4), PSL = photosynthesis, light reaction (L5), Cd = response to cold (L5), CO = chloroplast organization and biogenesis (L6), PSD = photosynthesis, dark reaction (L8), CMP = carotenoid metabolic process (L9). **GO cellular component**: Pd = plastid (L8), PP = plastid part (L9), TP = thylakoid part (L9), CPl = chloroplast (L9). **GO molecular function**: OR = oxidoreductase activity, acting on the CH-NH2 group of donors (L4), TF = translation factor activity, nucleic acid binding (L4). **KEGG**: CF = Carbon fixation in photosynthetic organisms.

#### Functional enrichment analysis of the PSY-ECG50

The high expression correlation of the *PSY-ECG50 *is a strong indicator that these genes may function in common biological processes. The *PSY-ECG50 *was therefore subjected to a functional enrichment analysis using "Fatigoplus" [33] which identified a number of significant enrichments in functional terms associated with the group (Table 1). In the biological process category, significant enrichments are found with genes associated with the terms photosynthesis, plastid organization and biogenesis, PQ biosynthetic process, and carotenoid and tetraterpenoid metabolic processes. In the cellular component category at level nine, genes associated with the terms plastid parts, thylakoid parts and chloroplasts are enriched.

Specifically, a number of genes in the *PSY-ECG50 *encode enzymes that directly function in the synthesis of chloroplastic localized isoprenoids. This includes *ZDS *(At3G04870) and *LCY-β *(At3G10230; r = 0.86 for both; Table 1, Figure 1) which function in the CrtBS pathway and the *PHYTOENE DESATURATION *2 (*PDS2*, At3g11950, r = 0.86) [3,34,35] and *ALBINO OR PALE GREEN MUTANT *1 (*APG1*; AT3G63410, r = 0.86) [36] genes that both function in the PQ biosynthesis pathway. In addition to its function as an electron carrier in PSII light-dependent photosynthesis reactions, PQ is also an essential compound in the synthesis of carotenoids where it has a role as a hydrogen acceptor in the desaturation reactions mediated by PDS and ZDS [3]. The *GLCYERALDEHYDE-3-PHOSPHATE DEHYDROGENASE B SUBUNIT (GAPDβ) *gene (At1g42970, r = 0.89) functions in the Calvin cycle to synthesize GAP which is a direct substrate for the MEP pathway [5,37].

In general, *PSY *is co-expressed with genes that encode proteins that have critical functional roles in the photosynthetic machinery; these proteins include enzymes that function in the biosynthesis of carotenoids, chlorophylls and components of the photosynthetic electron transport chain including PQ, PhQ, plastidial NAD(P)H dehydrogenase complex, thioredoxin, ferredoxin, plastocyanin and the cytochrome b6/f complex as well as proteins that form structural components of photosystem I and II. The high co-expression of *PSY *with genes that encode proteins that have important functional roles in the photosynthetic machinery, including a number of isoprenoid biosynthesis genes, illustrates that *PSY *is indeed highly co-expressed with functionally related genes and this gives confidence in the accuracy of the analysis.

#### PSY co-expression with functionally related isoprenoid biosynthesis genes

The expression correlation values were next extracted for all known Arabidopsis genes that encode enzymes that function in plastidial isoprenoid biosynthesis; this included Calvin cycle and MEP pathway genes as well as carotenoid, chlorophyll, PQ, PhQ, ABA, and GA biosynthesis genes (Figure 1 - see Additional File 1 for full list of genes). These genes will collectively be referred to as the *PSY*-correlated interrelated isoprenoid biosynthesis genes (*PSY-CIIG*).

This analysis revealed that the expression of all nuclear genes that encode enzymes that are known or predicted to function at each of the individual steps in the CrtBS pathway are highly correlated with *PSY *(Figure 1). In addition, *PSY *is also highly co-expressed with many isoprenoid-related biosynthesis pathway genes including, Calvin cycle and MEP pathway genes as well as chlorophyll, PQs and PhQs biosynthesis genes (Figure 1). It is noteworthy that the expression of *PSY *was found to be highly correlated with genes that function in different branches of chlorophyll biosynthesis; this includes *chlorophyll synthetase *(*ChlSyn*, At3g51820, r = 0.82) that functions in phytol side chain biosynthesis, as well as *glutamyl tRNA reductase *(*GluTR/HEMA1*, r = 0.77, e-value <1^-35^) and *glutamate 1-semialdehyde aminotransferase *(*GSA2*, r = 0.72, e-value <1^-35^) that function in the upstream tetrapyrrole branch of chlorophyll biosynthesis. Significantly, the *GluTR *and *GSA2 *genes encode enzymes that catalyze the biosynthesis of 5-aminolevulinic acid (ALA) which is the rate-limiting step for this pathway [38,39]. The high degree of co-expression of these genes strongly suggests that their transcription is regulated by a common mechanism. In contrast, none of the ABA biosynthesis genes that operate downstream of ABA1/ZEP, or any GA biosynthesis genes are positively expression correlated with *PSY *(Figure 1).

This analysis also revealed that for the carotenoid and chlorophyll biosynthesis-related pathway enzymes that are encoded by multiple genes, only specific family members displayed high co-expression levels; this may imply their functional importance in their respective biosynthesis pathways (Figure 1 and Additional File 1). In the MEP pathway, 1-deoxy-D-xylulose 5-phosphate synthase (DXPS) is the only enzyme that is encoded for by multiple (three) nuclear genes in Arabidopsis [40] and of these, only the functionally determined *DXPS2 *(At4g15560, r = 0.69, e-value <1^-35^) displays a high level of co-expression with *PSY *[41]. The two Arabidopsis IPP isomerase (IPPI) genes, show very little correlation with *PSY *(Additional File 1) and this is consistent with, and lends support to a recent study that reported that these enzymes have minor functional roles in plastidial isoprenoid biosynthesis, since IPPI and DMAPP are directly synthesized by the MEP pathway in plastids [42].

Of the family of 12 annotated *GGPPS *genes in Arabidopsis [41], only *GGPPS1 *(At4g36810; r = 0.74, e-value <1^-35^), that encodes a functionally active and plastid localized enzyme [43], displays a high level of expression correlation. The GGPPS-like protein, geranylgeranyl reductase (GGR, At4g38460, r = 0.64, e-value = 8.6^-35^) also shows some expression correlation, however, GGR does not have GGPPS activity *in vitro *and its function remains unknown although it has been suggested to encode a GPPS subunit [43].

Since GGPP is a common substrate, and thus an important metabolic link in the synthesis of multiple isoprenoid-derived compounds, it is significant that in addition to *PSY, GGPPS1 *is the most highly co-expressed *GGPPS *in Arabidopsis with a number of other genes that encode chloroplast localized enzymes that directly use GGPP as a substrate. These genes include *GGR2 *(At1g74470, *GGR2 *to GGPPS1, r = 0.53 (e-value = 2.2^-20^), data not shown) and two genes that encode solanesyl diphosphate synthase enzymes, *SPS-1 *(At1g78510, *SPS1 *to *GGPPS1*, r = 0.51 (e-value = 2.7^-18^), data not shown) and *SPS-2 *(At1g17050, *SPS2 *to *GGPPS1*, r = 0.58, (e-value = 6.8^-26^), data not shown). The GGR2 enzyme reduces GGPP to phytyl pyrophosphate [44] which forms essential phytol side chains for both chlorophyll and PhQ (vitamin K1) biosynthesis while the chloroplast localized SPS-2 enzyme catalyzes the synthesis of solanesyl diphosphate (SPP) which is thought to be a precursor of the PQ side-chain in Arabidopsis [45,46]. In addition, *PSY *is also highly co-expressed with all enzymes that function downstream in these pathways including, as mentioned *ChlSyn *for chlorophyll biosynthesis, *C-methyltransferase *for PhQ synthesis (At1g23360, r = 0.74) and, as mentioned above, *APG1 *and *PDS2 *for PQ synthesis (Figure 1).

**Figure 2:**
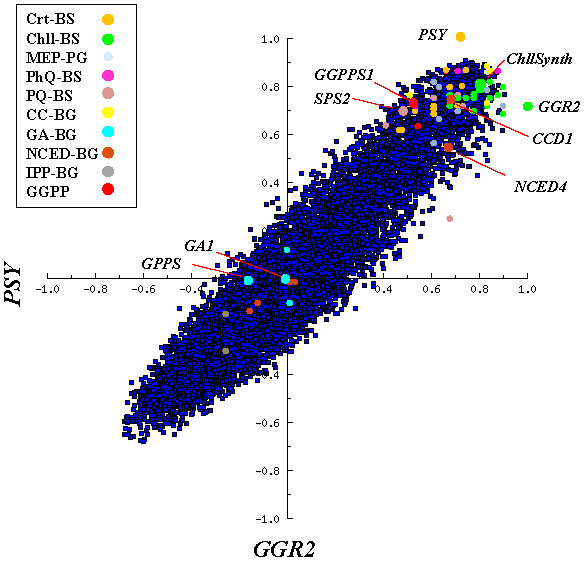
**Co-correlation scatter plot illustrating the level of co-expression of all Arabidopsis genes relative to *PSY *and *GGR2***. Genes that function in defined biosynthesis pathways (*PSY-CIIG *) are color highlighted as indicated in the legend. Select individual genes of interest are highlighted. All genes listed in Figure 1 are represented.

A co-correlation scatterplot between *PSY *and *GGR2 *(Figure 2) illustrates that both genes have a high level of co-expression with *GGPPS1 *and many genes that function in the chlorophyll, PQ and PhQ biosynthesis pathways thus providing strong evidence that the GGPPS1 enzyme plays a major role in generating a common pool of GGPP substrate that is used in the biosynthesis of these compounds. This interpretation is supported by a recent study that shows a reduction in carotenoid and chloroplast levels in a *ggpps1 *knock out mutant [47] and suggests that transcriptional regulation of the *GGPPS1 *gene serves as an important regulatory node in coordinating carotenoid, chlorophyll, PhQ and PQ biosynthesis.

The scatterplot also shows that expression of carotenoid and chlorophyll biosynthesis genes is not correlated with any of the plastid localized GA biosynthesis genes, and in particular, *GGPPS1 *showed no co-expression with *GA1*(r = -0.26, e-value = 0.04) or *GA2 *(r = - 0.03, e-value > 1), which directly use plastidial GGPP pools as substrates for GA synthesis. This implies that GGPPS1 does not function in the synthesis of GGPP for GA biosynthesis and this in turn is supported by reports that state that *ggpps1 *mutants show no signs of GA deficiency [47] and that the generation of GGPP pools for GA biosynthesis is dependent on the action of GPPS (At2g34630) [48].

**Figure 3:**
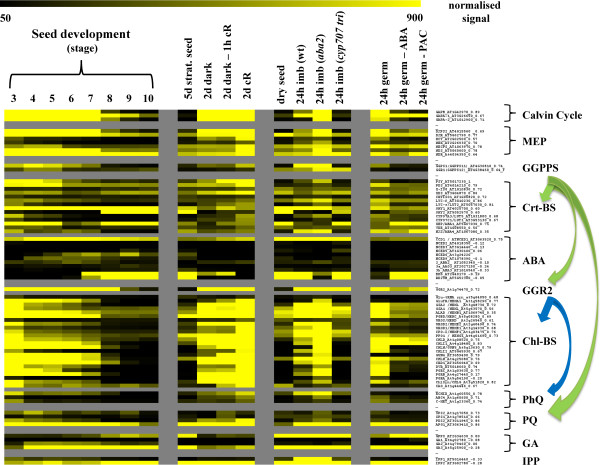
**Expression heat map illustrating the relative expression levels of the *PSY-CIIG *during early developmental stages. **The experimental conditions examined are listed across the top of the heat map and include, developing seed (GSE5634); stratified seed, etiolated and de-etiolated growth (GSE17159); imbibition in wild-type (wt), *aba2 *(ABA-deficient), and the *cyp707a1,a2,a3 *triple mutant (elevated ABA levels, GSE15700); post-germinative growth (24h) in the presence and absence of exogenously applied ABA or PAC (GSE5751). Individual genes included in the analysis are listed on the right and are arranged in sequential pathway order. Arrows indicate branch points where reaction products are used in multiple biosynthesis pathways. Results are presented as normalized signal values to reveal the relative expression levels of individual genes at conditions examined. Genes that function in biosynthesis pathway represented in Figure 1 are analysed. Details of the microarray experimental conditions are presented in Additional File 2 (Supporting Information).

Of the carotenoid cleavage dioxygenase gene (*CCD*) family, only *CCD1 *(At3g63520, r = 0.76, e-value <1^-35^), and to a lesser degree *CCD4 *(At4g19170, r = 0.51, e-value = 1.4^-18^), showed any degree of expression correlation with *PSY *(Additional File 1). In Arabidopsis, the CCD1 protein is localized to the cytoplasm and thus inaccessible to plastid localized carotenoid substrates [49]. It has been proposed to function in the metabolism of carotenoids that are localized in the chloroplast envelope [50] or present in dry seeds which lack well defined organelles such as chloroplasts [49]. Significantly, *PSY *is not co-expressed with any of the five plastid localized ABA biosynthesis *NCED *genes (-2, -3, -5, -6) that catalyze the synthesis of xanthoxin from β,β-xanthophylls [50,51], or other downstream enzymes that function in ABA biosynthesis (Figure 2 and 3), indicating that transcription of the CrtBS genes is not directly coupled to ABA biosynthesis. Indeed, previous reports have shown that ABA biosynthesis is correlated with the expression of the NCED genes suggesting that their expression is important in regulating ABA-biosynthesis [2].

### Stimulus specific expression analysis

The expression correlation analysis provides a generalized measure of how *PSY *is co-expressed with other genes in the genome since it is performed across multiple tissues and in response to a broad range of experimental conditions. In order to determine if expression of the genes corresponds to the known timing of carotenoid and chlorophyll biosynthesis and development of the photosynthetic apparatus, the expression of individual isoprenoid biosynthesis genes and genes in the *PSY-ECG50 *were examined throughout key developmental stages that are known to involve coordinated changes in the synthesis of carotenoids and chlorophylls.

Many previous studies on CrtBS gene expression have focused primarily on the transcriptional responses of a small subset of genes during de-etiolation since this is when large increases in CrtBS occur concomitant with the development of a functional photosynthetic apparatus [15,18,19,52]. However, carotenoids also have important functional roles during seed development, maturation and germination since they serve as precursors for ABA biosynthesis in developing seeds and CrtBS in dark-grown seedlings has been shown to be essential for PLB formation in etioplasts [14,17]. Thus, expression of the *PSY-CIIG *was examined throughout developmental stages encompassing seed development and maturation, imbibition and germination as well as etiolated and de-etiolated growth. Inhibitor and mutant experiments were also examined in order to determine the role that the early developmental-related phytohormones, ABA, GA and BR have in regulating the expression of these genes. Details of the experimental conditions for the microarray data examined are provided in Additional File 2.

#### Isoprenoid gene expression during seed and seedling development

The results in Figure 3 are presented as signal values since this provides information regarding the relative expression levels of individual genes at specific developmental stages and can provide insights into genes that may be rate-limiting due to low expression levels. While fold change ratios identify changes in expression in response to different conditions, they do not provide information of the relative abundance of transcripts.

The heat maps generated from the microarray expression analysis revealed that transcription of the *PSY-ECG50 *is modulated in a largely uniform manner in response to a range of different experimental conditions (Additional File 3) which is consistent with the high expression correlation of these genes. In general, the expression of *PSY*, other select CrtBS genes, chlorophyll, PQ and PlQ biosynthesis genes decline progressively throughout seed development, remained very low in dry and stratified seeds before being induced during imbibition, germination, skotomorphogenic and photomorphogenic growth (Figure 3). In addition, most genes that function in the upstream biosynthesis pathways to synthesize the commonly required GGPP precursor also follow a very similar expression profile, including the Calvin cycle *GAPD *subunit encoding genes (*GAPD -β*, A-1 and A-2); the *MEP *pathway genes *DXPS2, MCT *and *GGPPS1*. The very low expression level of specific genes in mature (stage 10), stratified and dry seeds may translate to very low protein/enzyme levels which would be rate-limiting for the biosynthesis of their respective molecules. While the activity of enzymes may be modulated to fine-tune biosynthesis rates, if there is no enzyme present then transcription of the gene will become the rate-limiting factor.

The observed induction in *PSY *expression in two day dark grown plants and in response to cR (Figure 3) in the absence of increased expression of *PDS *and *ZDS *is consistent with previous reports which additionally show that this response is sufficient to activate carotenoid biosynthesis [14,18,20]. The absence of a requirement for *PDS *and *ZDS *induction at these stages may be explained by the relatively high expression levels of these genes in mature (stage 10) and stratified seeds, when compared to *PSY*, which will presumably maintain relatively high enzyme levels. The activity of the PDS and ZDS enzymes however may be regulated by the abundance of the PDS2 enzyme; PDS2 functions in the biosynthesis of PQ [3,34] which is essential for the desaturation reactions mediated by PDS and ZDS [53]. Since the transcription of *PDS2 *closely mirrors that of *PSY *throughout these stages, it may be important in regulating the activities of the PDS and ZDS enzymes.

The collective moderate induction of these genes in dark grown plants and the strong induction in response to light coincide with the timing of carotenoid and chlorophyll biosynthesis during these developmental stages. As previously stated, carotenoids, the chlorophyll precursor Pchlide and the light-activated POR enzyme have been shown to accumulate in PLB of dark grown plants and carotenoid and chlorophyll biosynthesis is strongly activated in response to light [16]. This illustrates a correlation between biosynthesis pathway gene expression and carotenoid and chlorophyll biosynthesis at these developmental stages which implies that the transcriptional coordination of these pathway genes plays a major role in coordinating the synthesis of carotenoids and chlorophylls. Further, since *PSY *expression has been shown to be rate-determining and a major driving force for carotenoid biosynthesis [9,14], the closely coupled expression of other carotenoid and chlorophyll biosynthesis genes during these developmental stages suggests that expression of these genes may also be important in regulating and coordinating the biosynthesis of carotenoids and chlorophylls.

#### CrtBS gene expression and ABA biosynthesis in developing seeds

The expression profile of a number of CrtBS genes, including, *βCHY1 *and *-2 *and *ZDS/ABA1*, is in stark contrast with that of *PSY *during seed development in that their transcript levels remain elevated or increase during seed maturation, remain high in dry seeds and sharply decline during imbibition and in dark grown seedlings. These expression profiles are strikingly similar to a number of ABA biosynthesis genes, including NCED5, -6 and -9 that function directly downstream of *ZEP/ABA1*. Indeed, the induction of the ABA-responsive genes *EM6 *and *RD29B *[54] at latter stages of seed development (stage 7) strongly supports that an increase in endogenous ABA biosynthesis and accumulation occurs at this stage [55]. The coupled induction of *βCHY1 *and *-2*, and *ABA1 *with ABA biosynthesis genes during later stages of seed development that coincide with the accumulation of ABA indicates that *βCHY1 *and *-2*, and *ABA1 *may function to drive carotenoid intermediates towards β-xanthophyll and ultimately ABA biosynthesis during these stages. This is consistent with the observed reduced expression of *LCY-ε *in dry seeds and reports that *βchy1 βchy2 *double mutants have a reduced ability to synthesize ABA during drought stress [56]. Interestingly, while the expression of *βCHY1 *and *-2 *and *ABA1 *is reduced during imbibition and in dark grown plants, their expression is rapidly induced by light with *βCHY1 *being expressed at levels greater than two fold above that of *βCHY2*. The coupling of expression of these genes with other CrtBS genes in response to light may be indicative of their essential role in synthesizing β-xanthophylls which in turn are required for photoprotection.

#### Phytohormone regulation of isoprenoid gene expression in early development

Abscisic acid, GA and BRs have been shown to have important roles in regulating germination and post-germinative development and gene expression. Abscisic acid is known to inhibit germination and the expression of photosynthesis-related genes in imbibed seeds [54] while GA acts as essential hormone in promoting germination and etiolated development while negatively regulating ABA levels [57,58] in a process that is dependent on BRs [59]. Given the cross-talk between these important developmental-related phytohormones, we examined their role in regulating transcription of the genes using mutant and chemical treatment studies.

The induction of *PSY *and other photosynthesis-related genes following imbibition in continuous light is negatively regulated by ABA since in the ABA deficient mutant (*aba2*), induction is enhanced, while in the *cyp707a1, -a2 and -a3 *triple mutant, which has elevated ABA levels, the induction is almost completely abolished (Figure 3). ABA-mediated suppression is consistent with the known inhibitory role of ABA in germination and reports that ABA inhibits the expression of photosynthesis-related genes at this stage [54]. The observed ABA-mediated alteration of gene expression only occurs post-germination since gene expression levels in dry mutant seeds are not different from wild type (data not shown).

The presence of exogenous ABA or the GA biosynthesis inhibitor PAC in the growth media of light germinating seeds had very similar effects in that they strongly suppress the induction of *PSY *and other photosynthesis-related genes while maintaining expression of *βCHY2, ABA1 *and other ABA biosynthesis genes (Figure 3). The high expression level of *EM6 *and *RD29B *in PAC treated seeds indicates that these plants maintain high levels of ABA in the absence of GA. This is in line with reports that GA acts as essential hormone in promoting germination and negatively regulating ABA levels [57,58]. These results demonstrate that GA is required to activate the expression of *PSY *and the photosynthesis-related genes during germination in a process that is likely to involve a reduction in endogenous ABA levels. Indeed, ABA and GA are known to antagonistically regulate each others levels. GA levels in developing seeds follow an opposite trend to ABA in that they decrease progressively during seed maturation and increase sharply during germination [55].

The PAC-mediated repression of gene expression in light-germinated seeds observed here is in disagreement with a report that PAC increases expression of *PSY *and CrtBS genes in dark grown seedlings [14]. The discrepancy may be explained by differences in the growth conditions since in the above study [14], seeds were germinated in light for two to six hours before being grown in the dark for three days in the presence of PAC, whereas in the study analyzed here, seeds were stratified and germinated in the presence of light and PAC or ABA [60]. Thus, it appears that GA is essential for the early induction of these genes immediately following germination but may inhibit their expression at later developmental stages. This confirms reports that GA is required for germination and involved in the establishment of etiolated seedling development in darkness while repressing photomorphogenesis in a process that is dependent on BR [59].

**Figure 4:**
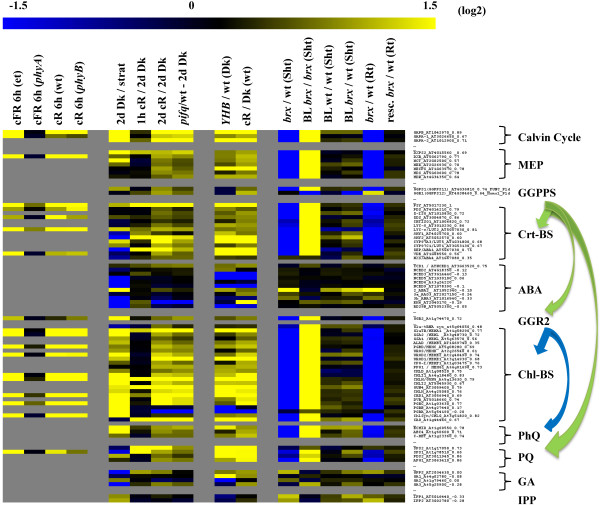
**Heat map illustration the fold change in expression of the *PSY-CIIG *in developmental-related mutants**. The experimental conditions included, etiolated and de-etiolated growth in *pifq *mutants (GSE17159), effect of constitutively active *PHYB*^*Y276H *^allele in dark-grown *phyAphyB *mutant seedlings (GSE8951), exposure of dark grown *phyA *and *phyB *mutants to cFR and cR light respectively (Tepperman 2001, 2004) and effect of brassinolide (BL) in roots (6 day) and shoots (4 day) of young *brx *loss-of-function plants (E-MEXP-635) Details of the microarray experimental conditions are presented in Additional File 2 (Supporting Information).

The BRs also appear to have a positive role in regulating the expression of the genes. The expression of the *PSY-CIIG *was strongly reduced to non-detectable levels in both root (six day old) and whole shoot tissue (four day old) in the *BREVIS RADIX *(*brx*) loss-of-function mutant; *brx *has an impaired root development phenotype due to a root-specific BR deficiency [61] (Figure 4). The transcription of the genes in *brx *was rapidly restored to control levels following three hour brassinolide (BL) treatment strongly indicating that the miss-regulated expression was due to BL deficiency [61]. The addition of BL to wild type plants, however, failed to alter expression of the genes, indicating that while optimal levels of BL are required for correct expression of the genes, excess BL does not induce further expression. In contrast to the reduced expression of photosynthesis-related genes in the 'minimal' shoot tissue of young *brx *seedlings [61], the shoot system morphology, including leaves of mature *brx *plants have been reported to resemble that of wild-type plants [62]. This suggests that the repression of photosynthesis-related genes in the shoot system of *brx *may only be temporary in young developing seedlings. The effect may result from ABA-mediated inhibition since BRs have been shown to positively regulate germination by reducing ABA sensitivity [63]. In addition, along with GA, BRs have been found to function in the establishment of etiolated development in Arabidopsis seedlings while repressing photomorphogenesis [59]. Thus, like GA, BL appears to be required for the normal expression of carotenoid and chlorophyll biosynthesis genes in young post-germination tissue in a process that may involve inhibition of ABA sensitivity and biosynthesis.

#### GA biosynthesis genes

The expression of *GPPS *that functions in GA biosynthesis, increased progressively during seed development, remained high in dry and imbibed seeds before being reduced in dark- and light-grown plants (Figure 3). This is consistent with the established role of *GPPS *in the synthesis of GGPP pools for GA biosynthesis, but not carotenoid and chlorophyll biosynthesis [48]. However, the *GA1 *and *GA2 *genes that encode plastid localized enzymes that directly use GGPP as a substrate showed minimal differential expression throughout. The expression profile of *GA3 *was similar to *GPPS *which is in line with their functional roles in the early steps in the GA biosynthesis pathway. The high expression level of *GPPS *in maturing, dry and imbibed seeds is likely to contribute to the increased synthesis of GGPP pools that is required for the increase in GA that occurs during germination [55,58]. The expression profile of these GA biosynthesis genes is in marked contrast to those of *GGPPS1, PSY *and *GGR2 *that decrease during seed maturation and increase in dark- and light-grown seedlings. Thus, these distinct expression profiles are entirely consistent with GPPS functioning in the synthesis of GGPP pools for GA biosynthesis and GGPPS1 catalyzing the synthesis of GGPP precursors for carotenoid and chlorophyll biosynthesis. A number of the late GA biosynthesis genes including the GA20 and GA3 oxidases that encode enzymes catalyzing the final steps in the synthesis of bioactive GAs are also strongly induced during imbibition (data not shown) in line with elevated GA levels that occur at this stage [58].

#### Regulation of isoprenoid gene expression by PHYs and PIFs during early development

The expression of the gene groups was next examined in a time course experiment where dark grown seedlings were exposed to cFR and cR. This experiment was performed on the 8 K *Arabidopsis *microarray chip that does not include all genes used in our analysis; it does however include *PSY *and a number of other genes under investigation and thus provides a reasonable representation of the biosynthesis pathways being examined [64,65]. It is not unexpected that exposure to both cFR and cR induced a largely universal increase in expression of the isoprenoid biosynthesis genes including, *PSY, GAPDβ, GGR2, GluTR, GSA2 *and *ChlSyn *(Figure 4, for 6 h time point). These light responses were additionally examined in *phy-A *and *-B *mutants since the PHYs are considered to be the predominant photoreceptors that mediate light-induced germination [66]. The cFR-induction was largely abolished for most genes in the *phyA *mutant while the cR-induction remained largely unaltered in the *phyB *mutant; these studies illustrate that *phyA *is required for early cFR-induced gene expression while the early cR induction can be mediated by PHYs other than PHYB. The importance of PHYA as an essential signaling component of cFR-regulated gene expression is well documented with a number of studies reporting that *phyA *mutants are disrupted in cFR-induced expression [64,65,67]. In addition, PHYA has been shown to be the dominant PHY in mediating the induction of early-response genes to cR [64,68,69] and to exert an early functional role in inhibiting hypocotyl growth [70]. However, while PHYB does not appear necessary to activate early cR-induced gene expression, *phyB *mutants have been reported to display a distinct morphological phenotype in cR, including long hypocotyls and small cotyledons, pointing to an important functional role for PHYB in plant photomorphogenesis [67].

While it is evident that cFR activates expression of many carotenoid and chlorophyll biosynthesis genes, and can induce de-etiolation (repress hypocotyl elongation) via a PHYA-dependent mechanism [67], it does not activate chlorophyll biosynthesis or chloroplast development which is dependent on light-induced activation of POR that catalyzes the conversion of Pchlide to chlorophyllide [22]. Indeed, it has been demonstrated that dark-grown seedlings exposed to cFR have carotenoid and chlorophyll contents that are around 80% and 20% respectively of the levels present in seedlings exposed to cR [18]. Further, cFR grown plants have a phenotype that is an intermediate between dark and cR grown plants [71] and cFR induces a PHYA-dependent growth pattern essential for soil emerging seeds or seedling survival in conditions of deep canopy shade which are characterized by reduced ratios of R:FR [72]. Hence, while expression of all three Arabidopsis POR genes (-A, -B and -C) is high in dark grown seedlings, the activation of their enzymatic activity and the induction of chlorophyll biosynthesis is ultimately light-dependent.

The PHYs are known to activate gene expression following their light-induced translocation from the cytoplasm to the nucleus where they specifically interact with PIFs and mediate their degradation [73,74]. The PIFs are a subset of basic helix-loop-helix (bHLH) transcription factors (TFs) that bind to the promoters of light-induced genes and function somewhat redundantly to repress their expression and photomorphogenesis in dark-grown seedlings [23,73]. The PHY-mediated degradation of PIFs allows activation of light-induced genes and *pif *mutants have been shown to have a *constitutive photomorphogenic (cop)-like *phenotype in true dark-grown seedlings [75]. A recent study showed that the PIF1 TF binds specifically to G-box *cis *motifs present in the *PSY *promoter and mutant studies revealed that PIF1 and other members of the PIF family function to inhibit *PSY *expression and carotenoid and chlorophyll biosynthesis in dark-grown seedlings [24]. The expression of the gene sets was thus examined in response to a number of PIF loss-of-function mutants in order to provide a broader systems perspective of the role that PIFs have in regulating the synthesis of chloroplast localized isoprenoid derived compounds.

The expression of the gene sets was not substantially altered in dark grown *pif *single and double mutants including *pif1, pif3 *and *pif4,5 *(data not shown) and this is probably a reflection of their redundant functions. In the *pif -1*,*-3*,*-4*,*-5 quadruple mutant (pifq)*, however, expression of the CrtBS genes and other genes involved in the synthesis of the photosynthetic apparatus reached quantitatively similar levels to that observed in 2 day cR-exposed wild-type plants (Figure 4). As reported previously for most dark grown *pifq *differentially expressed genes [23], the induction of the CrtBS genes occurs post-germination since their transcript levels are similar in *pifq *and wild type seeds. In this study [23], *PSY *and a number of other genes investigated, including *DXPS2, βCHY2, ABA1, GluTR, GUN5, chlorophyllide a oxygenase *(*CH1*) and *CHL1 *were identified as direct target candidates of PIF-mediated repression in the dark based on their expression being, firstly, elevated in dark grown *pifq *mutants compared to wild types, secondly, rapidly elevated after one hour Rc exposure (stimulates rapid PHY-induced PIF degradation ) and thirdly, sustained after germination in two days cR. In addition, 84% of genes in the *PSY-ECG50 *are induced >1.5 fold in dark-grown *pifq *mutants (Additional File 3).

While the PIFs clearly appear to negatively regulate expression of the genes in dark-grown seedlings, it is noted that expression of many genes including *PSY*, Calvin cycle genes, MEP pathway and chlorophyll biosynthesis genes (Figure 3) as well as most genes in the *PSY-ECG50 *(Additional File 3) were previously shown to be strongly induced in dark-grown wild-type seedlings when compared to stratified seeds. Although not as great as in response to light, for some genes, including, the three GAPD subunit encoding genes, *PSY, LCY-ε, DXPS2, MCT, GGR2, ChlSyn *and many other chlorophyll biosynthesis genes, the increase was greater than two-fold illustrating that expression of these genes is positively regulated in dark-grown wild-type seedlings. This documents that while the PIFs limit gene expression in dark grown seedlings, the inhibition is not absolute and that increases in expression do occur in the dark in the presence of PIFs. This is consistent with studies which show that increases in the biosynthesis of carotenoids and chlorophyll precursors in the dark is required for optimal greening upon light exposure [14].

In another related mutant experiment, dark-grown *phyA phyB *double mutant seedlings expressing the constitutively active Y^276^H missense allele of Arabidopsis PHYB (PHYB^Y276H^) [76], were similarly able to mimic the cR-induced transcriptional activation of the gene sets as observed in dark-grown *pifq *mutants. In this mutant, PHYB^Y276H ^undergoes light-independent nuclear localization which may mediate degradation of PIFs and allow the induction of light-inducible genes [77,78]. Thus, while the *phyB *mutant experiment indicates that cR induction of the gene sets can occur independently of PHYB, this experiment clearly shows that active and nuclear localized PHYB can induce expression of the genes in the dark.

In summary, these results strongly support that PHYA and PHYB have important functional roles in coordinating the transcription of the interrelated isoprenoid carotenoid and chlorophyll biosynthesis genes during de-etiolation. This process most likely involves the PHY-mediated degradation of PIFs, thus enabling light-induced gene expression.

#### Carotenoid gene expression and ABA biosynthesis in response to osmotic stress

**Figure 5:**
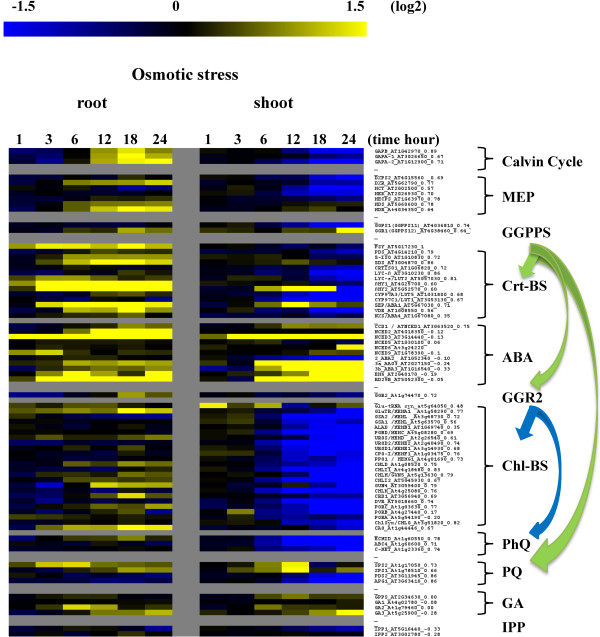
**Expression heat map time-course illustrating the effect of osmotic stress on expression of the *PSY-CIIG *in root and shoot tissue**. Fold-change (log2) in gene expression was measured in root and shoot tissue at the indicated time points following continuous osmotic stress application (mannitol) to root tissue (ME00327). Details of the microarray experimental conditions are presented in Additional File 2 (Supporting Information).

The carotenoids are precursors for ABA biosynthesis and we reported in this study that expression of some late CrtBS genes is induced at a time that coincides with increased ABA biosynthesis in maturing seeds (Figure 3). We therefore next examined expression of the *PSY-CIIG *in shoot and root tissue in a time course response to osmotic stress (mannitol) which induces the synthesis of ABA and can thus help resolve how expression of CrtBS genes is coordinated with that of ABA biosynthesis in these tissues. The experimental results reveal some interesting tissue specific expression response patterns (Figure 5). Not surprisingly, responses were more immediate in root tissue where the stress was applied, resulting in an early and sustained increase in expression of a number of the genes including, Calvin cycle genes, MEP pathway genes and dedicated CrtBS genes including, *PSY, ZDS, βCHY1 *and *-2, ABA1 *and *VDE*. In a similar manner to maturing seeds, this increase was paralleled with a strong increase in expression of a number of ABA biosynthesis genes including *NCED3*, and the ABA-responsive genes, EM6 and RD29B, suggesting an increase in endogenous ABA levels [79]. It is noted that there was little change in the expression of chlorophyll biosynthesis genes here.

The increase in expression of CrtBS pathway genes in root tissue is in contrast to that in the shoot where there is a general reduction in expression of carotenoid and chlorophyll biosynthesis genes from around 3-6 h which progressively decreases up to 24 h. However, there is a strong and transient induction of *βCHY2 *and *ABA1 *between 3-12 h in shoot tissue while *NCED3 *expression is induced early and sustained for the duration. As observed previously, *βCHY2 *and *ABA1 *expression is also strongly induced independent of other CrtBS genes during seed maturation, a process that also requires increased ABA biosynthesis and illustrates that expression of these genes can be uncoupled from other CrtBS genes, in both non-photosynthetic (seeds) and photosynthetic tissues, under conditions that require increased ABA biosynthesis.

The NCEDs have been proposed to be key regulators of ABA synthesis since their increased expression is correlated with increased endogenous ABA concentrations [80]. Notably here, *NCED2 *and *-3 *were the predominant *NCED*s induced in root and shoot tissue, which is in contrast to developing seeds where induction of the *NCED-5, -6*, and *-9 *genes parallels the increase in ABA production.

The more universal induction of carotenoid-related biosynthesis genes in osmotically stressed roots may reflect the lower concentration of carotenoid precursors that are present in this tissue. Since photosynthetic tissue contains high concentrations of epoxycarotenoids, it appears that transcription, and presumably translation of only the late CrtBS genes, *βCHY2 *and *ABA1 *are required to increase violaxanthin precursor levels for ABA biosynthesis. In contrast, in root tissue which is a major site of ABA biosynthesis, low concentrations of carotenoids may be rate-limiting for ABA biosynthesis. We therefore propose that an increase in Calvin cycle and MEP pathway genes and a more universal induction of the CrtBS genes is required in root tissue to generate violaxanthin precursors for ABA biosynthesis, a hypothesis that is supported by studies in maize [81] and rice [82]. The absence of any change in the expression of chlorophyll biosynthesis genes documents that the expression of the carotenoid and chlorophyll genes can be regulated independently, at least in root tissue.

### Promoter enrichment analysis

The high expression correlation values of the genes within the *PSY-ECG50 *points to the possibility that their expression is coordinately regulated. A promoter content analysis was therefore performed in an attempt to identify the presence of enriched putative regulatory elements that may be causative for their co-expression. The analysis was performed examining regions 2000 base pairs (bp) upstream of the coding region/translation start sites (TlSS) of genes since regulatory elements have previously been identified in 5 prime untranslated regions (5' UTR) of light-induced genes [83,84]. This is particularly relevant to *PSY *which is annotated in TAIR to have a 779 bp sequence upstream of the coding region that includes two 5'UTRs and an intron. A number of elements were found to be significantly enriched in the promoters of the co-expressed *PSY-ECG50 *and are thus considered candidate regulatory elements that may coordinate their transcription. In addition, a number of these elements correspond to known plant *cis *regulatory elements including, a slightly degenerate G-box (CACGNG (p-value = 9.8^-03^) compared to CACGTG) and the auxin-responsive element (AuxRE, TGTCTC (p-value = 0.02), Additional File 4).

The G-box is known to be present in the promoters of many light-regulated genes [85-87] and is enriched in the promoters of genes that are rapidly-induced by PHYA [88]. As previously mentioned, G-boxes present in the promoter of *PSY *have been shown to specifically bind the PIF1 TF resulting in inhibition of *PSY *expression [24]. Thus, the identification of an enrichment of G-boxes in the promoters of the *PSY-ECG50 *is consistent with the observed PHYA dependency for induction of these genes and the inhibitory effect of PIFs on their expression in dark grown plants. We noted that one of the two G-boxes identified in the PSY promoter in this analysis is positioned in the 5' UTR in close proximity to the TlSS (-21 to -16 and -919 to -914) and both differ from those identified previously [67] which examined promoters more than 2000 bp upstream of the TlSS.

The enrichment of AuxREs in the promoters of the genes is consistent with the observed BR-dependency for CrtBS gene expression in young tissues. While the AuxRE was initially believed to confer auxin responsiveness to promoters [89,90], more recent studies have indicated that this element is also a target of BR signaling. It has been suggested that the AuxRE should in fact be considered a BR-AuxRE [91] since it has been found to be enriched in auxin- and BR-responsive genes rather than genes specifically regulated by auxin [92]. Thus, the enrichment of BR-AuxRE in the promoters of the genes is in line with the BR-dependent gene expression observed in young tissues in this study and additionally adds strength to studies that have shown BRs have a role in the establishing the etiolated development program in dark-grown Arabidopsis seedlings [59].

In summary, the GA- and BR-dependent induction of the carotenoid and chlorophyll biosynthesis genes following germination is entirely consistent with the role of these hormones in establishing etioplast development [59] and the requirement for carotenoid and chlorophyll precursor accumulation in developing etioplasts [14]. The expression level of the genes appears to be restricted by the PIF TFs in dark grown plants which are subsequently degraded by light activated PHY molecules allowing a strong and coordinated induction of the genes and a subsequent increase in carotenoid and chloroplasts biosynthesis. The identification of an enrichment in putative BR-AuxRE and G-boxes in promoters of the *PSY-ECG50 *complements the observed transcriptional regulatory roles of BRs and PIFs respectively.

## Conclusions

The tightly coupled expression and induction of PSY and many other isoprenoid biosynthesis genes throughout key developmental stages that correspond to the timing of increased carotenoid and chlorophyll synthesis and development of the photosynthetic apparatus strongly suggests that the coordinated transcription of these biosynthesis genes is critical in regulating and coordinating the biosynthesis of the functionally related carotenoid, chlorophyll, PQ and PhQ molecules. The phytohormones GA, BR and ABA as well as the transcriptional-related PHYs and PIFs appear to have important roles in regulating and coordinating the transcription of these isoprenoid-derived compounds.

## Methods

### PSY expression correlation analysis

An expression correlation analysis was performed for PSY using the freely available Arabidopsis co-expression tool (ACT) (http://www.arabidopsis.leeds.ac.uk/)[31]. This particular tool uses hybridization signal intensities from microarray experiments to calculate a Pearson correlation coefficient (r-value), which is a scale-invariant measure of expression similarity. The analysis was performed across all of the 322 available Ath1 22 K microarrays from the NASC/GARNet dataset which contain probe sets that recognize 21,891 Arabidopsis genes. The arrays included in this analysis are derived from a broad range of experimental samples including specific tissue types, developmental stages, abiotic and biotic treatments, and a range of mutants. Importantly, the ACT tool uses NASC/GARNet data sets that were labeled, hybridized and analyzed using a standardized procedure thus providing a homogeneous and readily comparable data set.

The analysis was performed leaving the gene list limit blank resulting in the return of a global correlation analysis of all probe sets relative to PSY ranging from the most positive to the most negatively expression correlated genes (total over 22,500 probe IDs). The top 50 genes that had the highest expression correlation with PSY were extracted from the list as were genes that were included in the *PSY-CIIG*. Both lists were filtered to include only genes that were represented by a unique probe on the microarray chip.

The co-correlation analysis was performed using the 2D scatter plot tool present on the ACT website. Probe IDs for *PSY *and *GGR2 *was inserted into the X and Y axis and all other specifically highlighted genes were inserted in the highlight option.

### Functional enrichment analysis

A gene ontology (GO) analysis was performed using the "Fatigo plus" (version 3.1) compare tool in the Babelomics suite (http://babelomics.bioinfo.cipf.es/functional.html) [33,93] to determine if there was any statistically enriched terms associated with the *PSY-ECG50 *expression correlated genes compared to the expected frequency in the complete genome. The top 50 genes were selected for this analysis since their expression was highly correlated with *PSY *(r-value range 0.91-0.84). All the available functional annotation options for Arabidopsis were selected which include the three GO categories of biological process (BP), cellular component (CC) and molecular function (MF) as well as KEGG pathways. The tool uses a Fisher's exact test and returns adjusted *p*-values (Family Wise Error Rate) to accounting for multiple testing to determine statistical significance.

### Microarray stimuli specific transcription analysis

An *in silico *global expression analysis was subsequently performed for both gene sets in response to specific stimuli and in selected mutants to identify conditions that induce differential expression of the genes (Figures 3 and 5 and Additional File 3). The expression profiles of *PSY *and its positively correlated gene sets were initially screened over all of the available ATH1: 22 K array Affymetrix public microarray data in the gene response viewer tool (GRV) in Genevestigator [94]. Normalized microarray data were downloaded for experiments that were found to induce differential expression of the genes from the following sites:

NASCArrays (http://affymetrix.arabidopsis.info/narrays/experimentbrowse.pl)[95], TAIR-ATGenExpress (http://www.ebi.ac.uk/microarray-as/ae/), GEO (NCBI) (http://www.ncbi.nlm.nih.gov/geo/) [96].

(see attached file for experiment descriptions).

### Promoter enrichment analysis

A number of tools in the POXO (http://ekhidna.biocenter.helsinki.fi/poxo) [97] promoter analysis suite were used to analyze promoter regions 2000 bp upstream of the coding regions of the genes in the *PSY-ECG50*. The POCO tool was used to identify enriched elements and the POBO tool was used to verify the presence of identified elements in the PSY promoter. The identified significantly enriched motifs were filtered to ensure that they were present and enriched in the PSY promoter and were present in greater than 70% of the genes in the *PSY-ECG50*.

## Authors' contributions

ETW and CG conceived the initial project. OT and RV performed an initial analysis on the carotenoid biosynthesis pathway genes and promoters. SM expanded the project to include additional isoprenoid biosynthesis pathway genes, generated the results presented, interpreted data and wrote the manuscript with contributions by ETW, OT and CG. All authors read and approved the final manuscript.

## Supplementary Material

Additional file 1 **Additional Table 1**. Extended list of genes in the *PSY*-correlated interrelated isoprenoid biosynthesis genes and their expression correlation relative to PSY. Click here for file

Additional file 2 **Additional Text 1**. Description of experimental conditions in the microarray data-sets examined. Click here for file

Additional file 3 **Additional Figure 1**. Heatmaps illustrating the expression of the *PSY-ECG50 *in response to the range of experimental conditions examined. Click here for file

Additional file 4 **Additional Table 2**. Enriched motifs identified in the promoters of genes in the *PSY-ECG50*. Click here for file
