# Supplementary material for: A transcriptional analysis of carotenoid, chlorophyll and plastidial isoprenoid biosynthesis genes during development and osmotic stress responses in Arabidopsis thaliana
Source: BMC Syst Biol. 2011 May 19;5:77. doi: 10.1186/1752-0509-5-77 (PMC3123201; doi:10.1186/1752-0509-5-77)
Supplement: Additional file 2 — Additional Text 1. Description of experimental conditions in the microarray data-sets examined. [file 1752-0509-5-77-S2.DOC]

**EXPERIMENT DESCRIPTIONS**

**Seed & silique development (GSE5634)**

RNA was extracted from seeds of 8 week old, long day (16/8) soil grown plants

stage 3: siliques, w/ seeds, mid globular to early heart embryos; stage 4: siliques, w/ seeds, early to late heart embryos; stage 5: siliques, w/ seeds late heart to mid torpedo embryos, stage 6:seeds w/o siliques, mid to late torpedo embryos, stage 7: seeds w/o siliques, late torpedo to early walking-stick embryos; stage 8: seeds w/o siliques; walking-stick to early curled cotyledons embryos, stage 9: seeds w/o siliques; curled cotyledons to early green cotyledons embryos; stage 10, seeds w/o siliques, green cotyledons embryos.

Genotype (Col-0)

***pifq* expression during stratification, dark growth and cR (GSE17159)**

Seeds were exposed to white light (WL) for a total of 1.5 hours after imbibition. They were then stratified for 5 days at 4ºC in darkness, induced to germinate with a 5-min red pulse (Rp) and then incubated in the dark for 3h at 21°C before exposure to a terminal 5-min far red pulse (FRp) to suppress pseudo-dark effects. Seeds were then placed in either dark (D) or constant red light (Rc) (6.7 μmol/ m2/s) at 21°C for 45h (2d-old seedlings). Alternatively, 2d-old dark-grown seedlings were treated with 1h of red light (R1) (7.5 μmol/m2/s). Seed samples were harvested after stratification (5d stratified seeds).

Wild-type (Col-0) and *pif1pif3pif4pif5* mutants

**Expression in dry and imbibed seeds (GSE15700)**

Freshly harvested seeds were imbibed in water for 24h under continuous light.

Genotypes: Wild type (Col-0), *aba2-1* and *cyp707a1-1a2-1a3-1* triple mutants.

**Post-germinative transcriptome in the presence of ABA and PAC (GSE5751)**

Seeds were sown on MS medium supplemented with 20uM ABA, 20uM paclobutrazol (PAC), or nothing, stratified for 3 days at 4 4°C, then transferred to a growth room at 22°C under continuous white light at 80-100 µmol m-2 for 24 hours

Genotype: Ler-0

**De-etiolation time course cFR-*phyA* and cR-*phyB* (Tepperman 2001, 2004)**

Arabidopsis seeds were stratified for 5 days at 4°C, exposed to white light for 2 h to induce germination and placed in a growth chamber at 21°C in total darkness for 4 days. Seedlings were then irradiated with cFR (740 nm, 2 µmol m^-2^ sec^-1^ -*phyA*), or cR (660 nm, 7 µmol m^-2^ sec^-1^ -*phyB*) and tissue was harvested and frozen immediately in liquid nitrogen at 1, 3, 6, 12, 18, and 24 h after the start of irradiation

Genotypes Wild-type Arabidopsis (ecotype RLD), phyA-101 and phyB seeds

**Light independent allele of phytochrome B (GSE8951)**

Comparative transcript profiling experiments were performed to assess whether the pattern of gene expression regulated by dominant gain-of-function alleles of Arabidopsis phytochrome B accurately reflects the process of photomorphogenesis in wild-type Arabidopsis. Three genotypes were grown in darkness for 4 days in this study including, wild type (Ler), the *phyA-201phyB-5* double mutant and *YHB* (*AtPHYB-YH/phyA-201phyB-5*). Additionally, wild type lines were grown under continuous red light (Rc) for four days at 15 µmol m-2 s-1 Rc and 50 µmol m-2 s-1 Rc and YHB was also grown under 50 µmol m-2 s-1 Rc.

(all dark treatments n=3, light treat n=2)

**Arabidopsis *brx* lines (E-MEXP-635)**

Six days after germination roots were excised and RNA was extracted.

Four days after germination in liquid media, seedlings were either mock-treated or treated with 1mM IAA for 1 hour or with 5nM brassinolide for 3 hours.

Genotypes: Slavice-0 (Sav-0), loss-of-function *brx* line, and rescued *brx*.

**Osmotic stress (ME00327)**

This experiment studied the effects of continuous osmotic stress applied to root tissue on gene expression in root and shoot tissue. Plant material from 18 days old Arabidopsis thaliana plants was harvested at the following time points: 0,5h; 1h; 3h; 6h; 12h; 24h following stress application. For each time point two samples serving as replicates were collected. Root and shoot material was separated and handled individually. Control samples were collected from non-treated plants at the respective time points.

Genotype: Columbia-0
