# Supplementary material for: A transcriptional analysis of carotenoid, chlorophyll and plastidial isoprenoid biosynthesis genes during development and osmotic stress responses in Arabidopsis thaliana
Source: BMC Syst Biol. 2011 May 19;5:77. doi: 10.1186/1752-0509-5-77 (PMC3123201; doi:10.1186/1752-0509-5-77)
Supplement: Additional file 3 — Additional Figure 1. Heatmaps illustrating the expression of the PSY-ECG50 in response to the range of experimental conditions examined. [file 1752-0509-5-77-S3.PPT]

## Slide 1
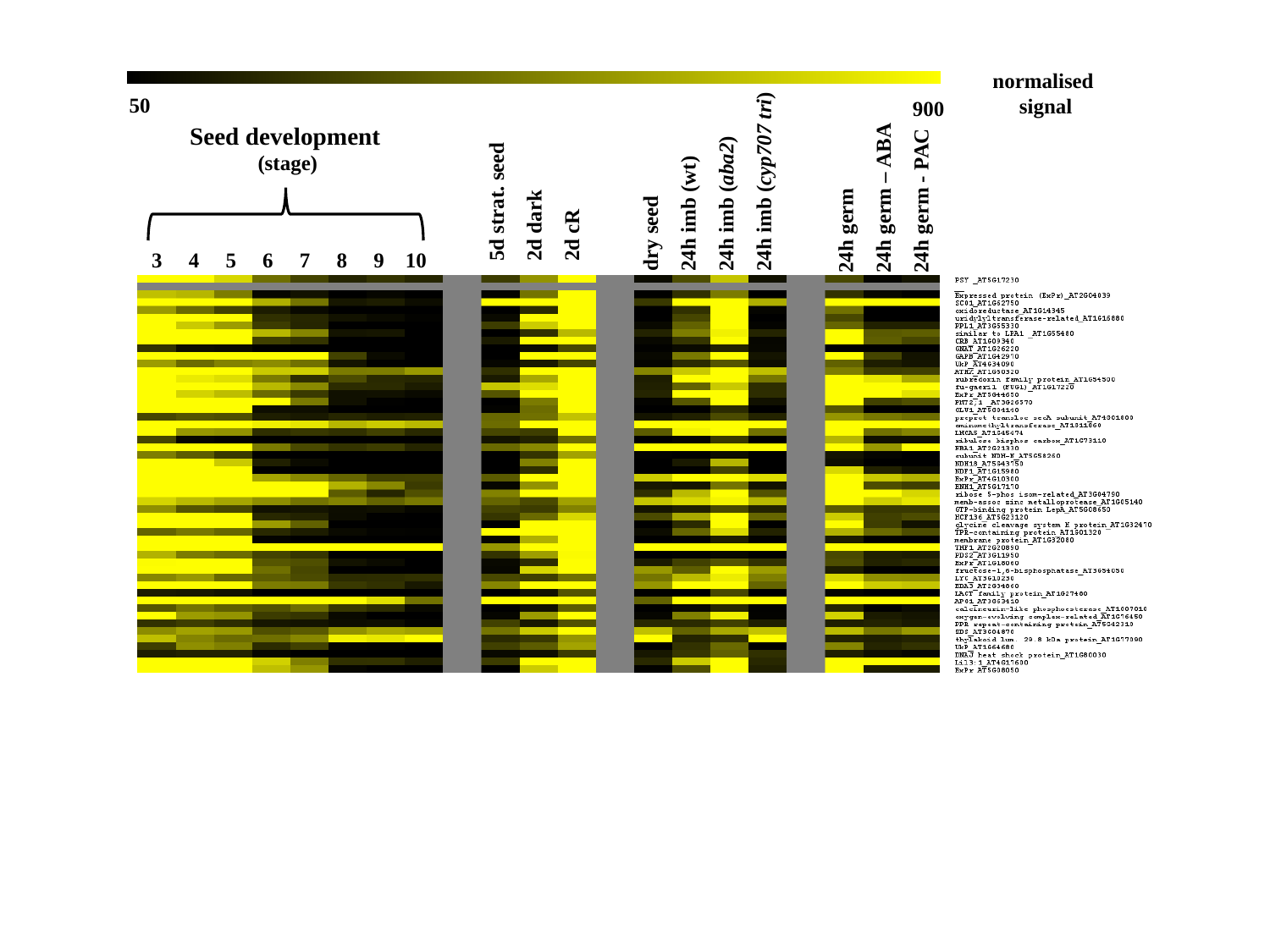

normalised
signal
50
900
dry seed
24h imb (wt)
24h imb (aba2)
24h imb (cyp707 tri)
Seed development
 (stage)
24h germ
24h germ – ABA
24h germ - PAC
5d strat. seed
2d dark
2d cR
3 4 5 6 7 8 9 10

## Slide 2
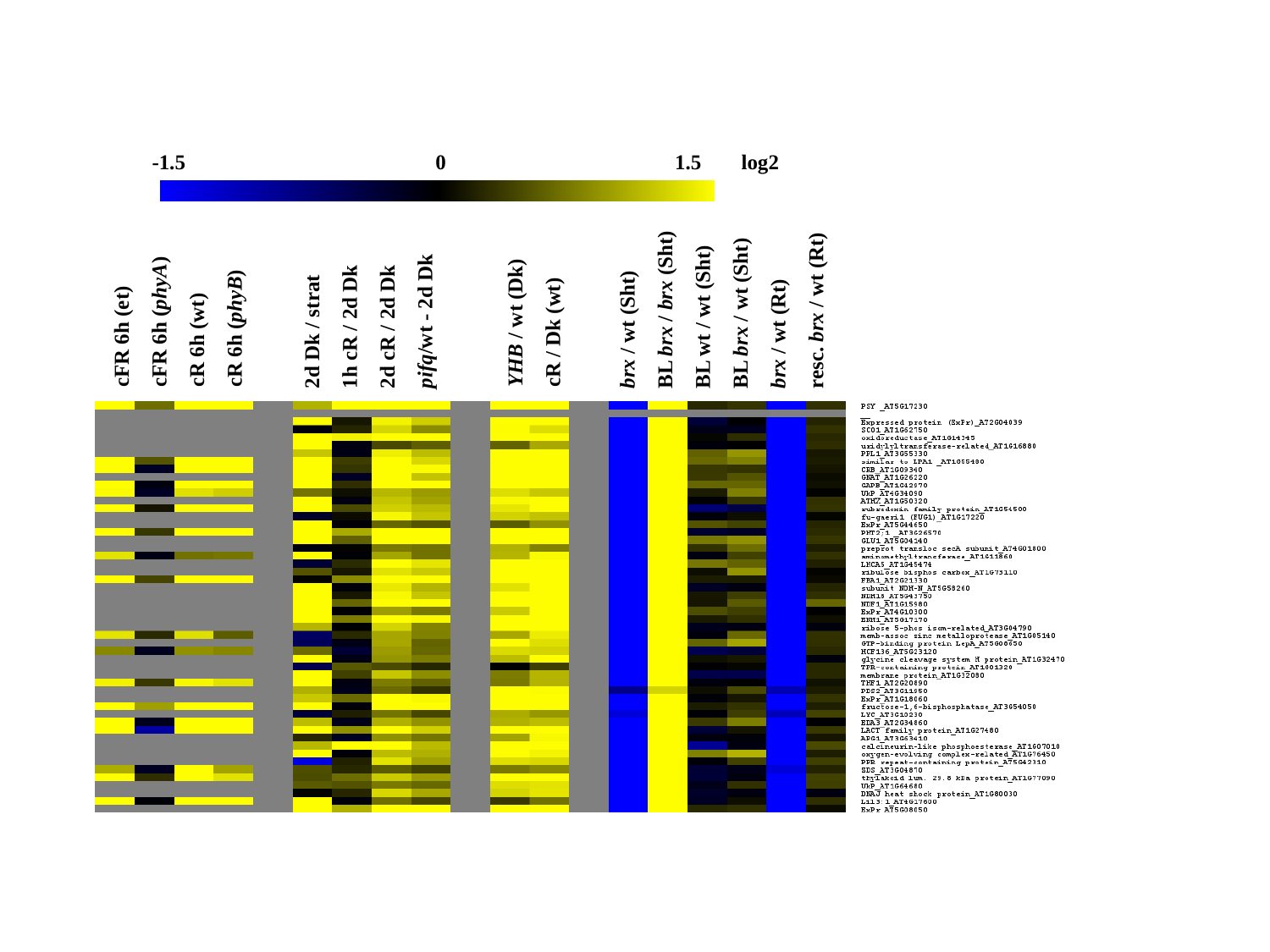

-1.5
0
1.5
log2
brx / wt (Sht)
BL brx / brx (Sht)
BL wt / wt (Sht)
BL brx / wt (Sht)
brx / wt (Rt)
resc. brx / wt (Rt)
2d Dk / strat
1h cR / 2d Dk
2d cR / 2d Dk
pifq/wt - 2d Dk
cFR 6h (et)
cFR 6h (phyA)
cR 6h (wt)
cR 6h (phyB)
YHB / wt (Dk)
cR / Dk (wt)

## Slide 3
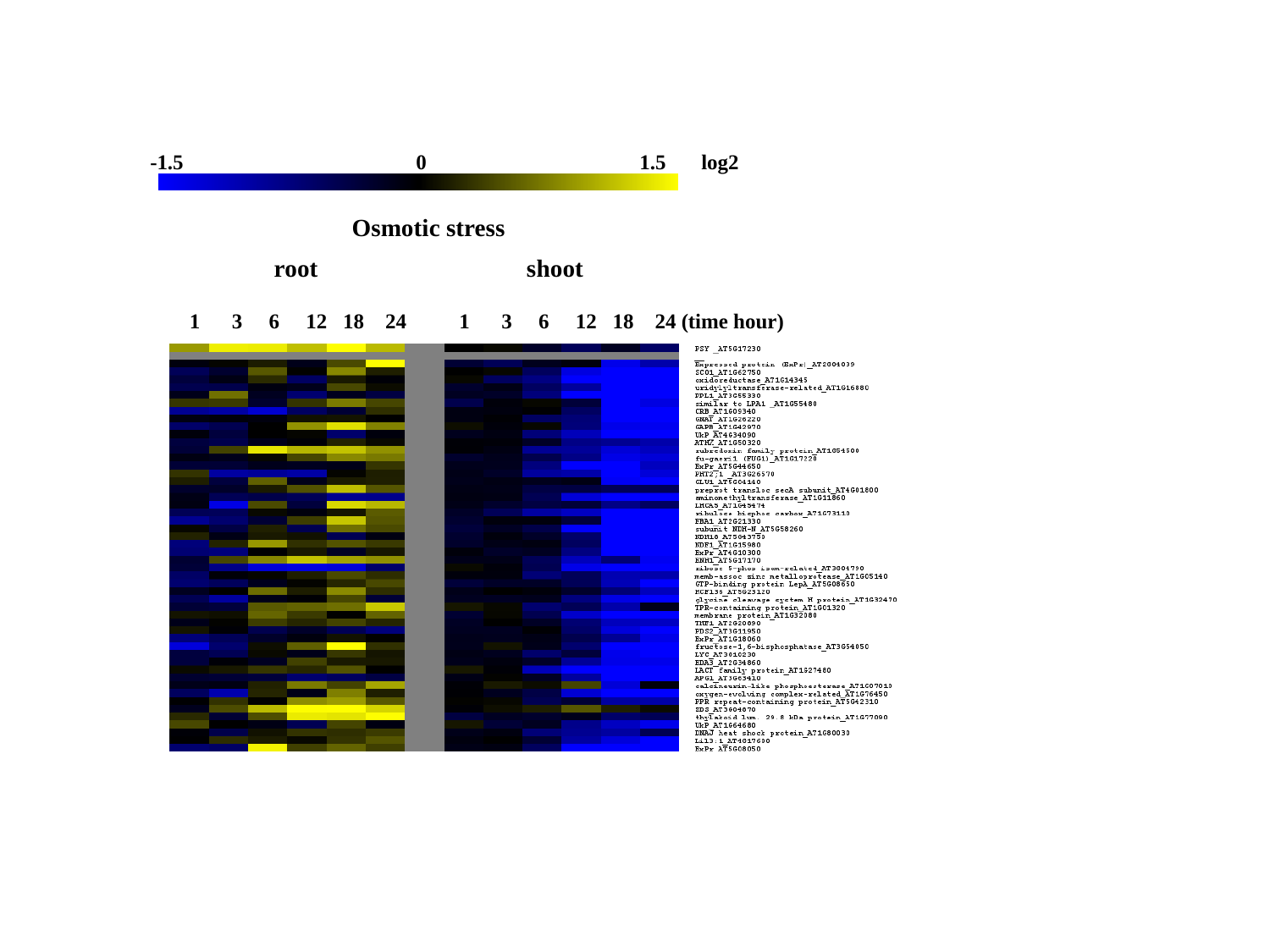

-1.5
0
1.5
log2
Osmotic stress
root shoot
1 3 6 12 18 24 1 3 6 12 18 24 (time hour)
