# Supplementary material for: A transcriptional analysis of carotenoid, chlorophyll and plastidial isoprenoid biosynthesis genes during development and osmotic stress responses in Arabidopsis thaliana
Source: BMC Syst Biol. 2011 May 19;5:77. doi: 10.1186/1752-0509-5-77 (PMC3123201; doi:10.1186/1752-0509-5-77)
Supplement: Additional file 4 — Additional Table 2. Enriched motifs identified in the promoters of genes in the PSY-ECG50. [file 1752-0509-5-77-S4.DOC]

|  |  |  | **COPIES / PROMOTER** | | | **% PROM contain** | |  |
| --- | --- | --- | --- | --- | --- | --- | --- | --- |
| **Enriched pattern** | **Match plant TF** | **consensus** | **PSY** | **PSY50**  **(av)** | **BG**  **(av)** | **PSY50** | **BG** | ***p value*** |
| ATACGA |  |  | 2 | 1.09 | 0.67 | 70.6 | 47.7 | 1.54E-04 |
| TCTCAC |  |  | 5 | 1.46 | 1.02 | 76.5 | 62.0 | 2.72E-03 |
| TCCTCT |  |  | 3 | 2.16 | 1.52 | 90.2 | 73.2 | 2.64E-04 |
| CACGNG | G-box | CACGTG | 2 | 1.86 | 1.32 | 76.5 | 59.8 | 9.80E-03 |
| ACAAGT |  |  | 3 | 2.15 | 1.58 | 86.3 | 77.4 | 4.39E-03 |
| GAGCNC |  |  | 2 | 1.74 | 1.29 | 76.5 | 58.5 | 2.82E-02 |
| AGGAAG |  |  | 4 | 1.87 | 1.40 | 76.5 | 70.3 | 1.33E-02 |
| GAGAAT |  |  | 2 | 2.33 | 1.75 | 94.1 | 80.7 | 1.53E-03 |
| TTNGGC |  |  | 5 | 3.46 | 2.62 | 100 | 90.3 | 1.98E-04 |
| AGAAGA |  |  | 6 | 5.49 | 4.21 | 98.0 | 94.7 | 2.57E-03 |
| TCTTCC |  |  | 5 | 2.22 | 1.71 | 80.4 | 77.4 | 1.08E-02 |
| TANGCC |  |  | 2 | 1.89 | 1.46 | 84.3 | 74.4 | 1.19E-02 |
| GCNCAA |  |  | 3 | 3.11 | 2.40 | 98.0 | 88.4 | 1.38E-03 |
| TGTCTC | ARF | TGTCTC | 3 | 1.50 | 1.16 | 74.5 | 66.1 | 2.09E-02 |
| CNCTCA |  |  | 4 | 3.33 | 2.58 | 96.1 | 90.0 | 4.03E-04 |
| CTCTTC |  |  | 4 | 2.48 | 1.93 | 88.2 | 79.9 | 2.15E-02 |
| GNGCNC |  |  | 4 | 3.69 | 2.91 | 82.4 | 71.4 | 3.04E-02 |
| TCNCTC |  |  | 6 | 5.83 | 4.60 | 98.0 | 95.4 | 1.36E-02 |
| TCTCNC |  |  | 9 | 6.15 | 4.87 | 100 | 95.5 | 9.03E-03 |
| CAAGTT |  |  | 2 | 2.12 | 1.68 | 92.2 | 80.1 | 3.10E-02 |
| TGATTC |  |  | 3 | 2.25 | 1.79 | 84.3 | 80.8 | 3.91E-02 |
| AGANGA |  |  | 12 | 10.68 | 8.53 | 100 | 99.6 | 2.06E-04 |
| CAAACT |  |  | 4 | 2.34 | 1.87 | 92.2 | 84.1 | 1.41E-02 |
| GAAGAA |  |  | 10 | 5.15 | 4.19 | 96.1 | 94.7 | 1.32E-02 |
| TGCTNT |  |  | 5 | 5.24 | 4.28 | 96.1 | 97.4 | 4.06E-03 |
| TNGCCA |  |  | 5 | 2.97 | 2.42 | 94.1 | 86.5 | 2.55E-02 |
| CTTCNT |  |  | 12 | 9.55 | 7.87 | 100 | 99.4 | 1.50E-03 |
| GAAAAA |  |  | 8 | 5.57 | 4.68 | 100 | 97.7 | 5.94E-03 |
| GAAGANAA |  |  | 4 | 2.20 | 1.28 | 80.4 | 69.8 | 5.81E-08 |
| TTTTNTTC |  |  | 5 | 3.23 | 2.11 | 96.1 | 83.6 | 1.98E-07 |
| CNCTCAA |  |  | 2 | 1.39 | 0.91 | 82.4 | 58.1 | 7.73E-05 |
| AGNAAGNG |  |  | 4 | 1.87 | 1.26 | 84.3 | 67.8 | 8.98E-04 |
| CNTNCTCT |  |  | 3 | 2.17 | 1.49 | 82.4 | 73.3 | 3.53E-04 |
| ANGNTGAG |  |  | 2 | 1.59 | 1.10 | 82.4 | 63.3 | 3.56E-04 |
| AGAAAAA |  |  | 7 | 3.11 | 2.18 | 100 | 84.2 | 1.65E-05 |
| AGTNTTC |  |  | 5 | 1.90 | 1.34 | 84.3 | 73.6 | 5.94E-04 |
| TTNTCTNC |  |  | 5 | 3.66 | 2.60 | 94.1 | 91.2 | 7.36E-07 |
| GANTCAA |  |  | 4 | 2.55 | 1.82 | 94.1 | 80.4 | 2.18E-04 |
| TNGATTC |  |  | 4 | 2.29 | 1.69 | 92.2 | 79.3 | 1.90E-03 |
| GTNTTCT |  |  | 3 | 2.62 | 2.00 | 92.2 | 85.3 | 1.48E-03 |
| GAAGANA |  |  | 8 | 4.11 | 3.25 | 94.1 | 93.9 | 1.47E-03 |
| ANCCAAA |  |  | 6 | 3.84 | 3.10 | 100 | 94.1 | 1.02E-03 |

NOTE:

PSY = PSY promoter only

PSY50 = average in promoters of the PSY-ECG50
